# Path Sampling Simulations Reveal How the Q61L Mutation Alters the Dynamics of KRas

**DOI:** 10.1021/acs.jpcb.2c06235

**Published:** 2022-11-25

**Authors:** Sander Roet, Ferry Hooft, Peter G. Bolhuis, David W. H. Swenson, Jocelyne Vreede

**Affiliations:** † Van’t Hoff Institute for Molecular Sciences, University of Amsterdam, Science Park 904, 1098 XHAmsterdam, The Netherlands; ‡ Department of Chemistry, Norwegian University of Science and Technology (NTNU), NO-7491Trondheim, Norway; § Laboratoire de Physique and Centre Blaise Pascal, CNRS, Univ Lyon, ENS de Lyon, Univ Claude Bernard, 69007Lyon, France

## Abstract

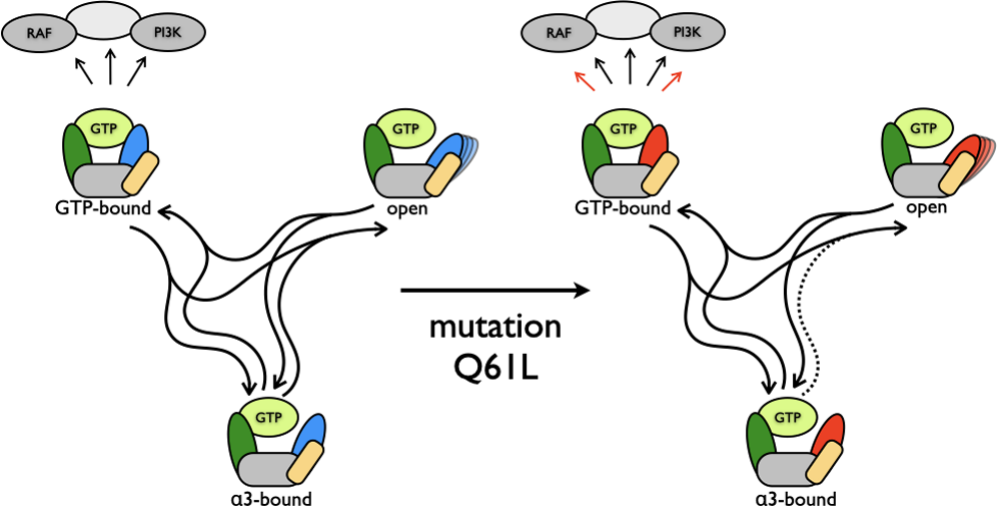

Flexibility is essential for many proteins to function, but can be difficult to characterize. Experiments lack resolution in space and time, while the time scales involved are prohibitively long for straightforward molecular dynamics simulations. In this work, we present a multiple state transition path sampling simulation study of a protein that has been notoriously difficult to characterize in its active state. The GTPase enzyme KRas is a signal transduction protein in pathways for cell differentiation, growth, and division. When active, KRas tightly binds guanosine triphosphate (GTP) in a rigid state. The protein–GTP complex can also visit more flexible states, in which it is not active. KRas mutations can affect the conversion between these rigid and flexible states, thus prolonging the activation of signal transduction pathways, which may result in tumor formation. In this work, we apply path sampling simulations to investigate the dynamic behavior of KRas-4B (wild type, WT) and the oncogenic mutant Q61L (Q61L). Our results show that KRas visits several conformational states, which are the same for WT and Q61L. The multiple state transition path sampling (MSTPS) method samples transitions between the different states in a single calculation. Tracking which transitions occur shows large differences between WT and Q61L. The MSTPS results further reveal that for Q61L, a route to a more flexible state is inaccessible, thus shifting the equilibrium to more rigid states. The methodology presented here enables a detailed characterization of protein flexibility on time scales not accessible with brute-force molecular dynamics simulations.

## Introduction

For many proteins, their flexibility is essential for their function. For example, receptor proteins undergo a conformational change upon detection of a signal, and change back to their inactive form once the trigger is gone. Also, further along in a signal transduction pathway, the proteins involved undergo conformational changes when upon activation, and subsequent deactivation. These changes are often subtle, which makes them very difficult to characterize, as experiments lack the required resolution in time, space, or both. In this work, we focus on a protein that has been notoriously difficult to characterize in its active state. Ras GTPases are signal transduction proteins that mediate cell growth, cell differentiation, and death. Binding of guanosine triphosphate (GTP) activates signal transduction by Ras proteins, while their GTPase function inactivates signal transduction again by hydrolyzing GTP to guanosine diphosphate (GDP). Ras GTPases comprise the most frequently occurring family of oncoproteins in human cancers.^1,2^ Mutations in Ras proteins initiate cell transformation, drive oncogenesis, and promote tumor maintenance. The Ras family of oncoproteins has been studied extensively for almost three decades, as activation of Ras represents a key feature of malignant transformation for many cancers. In the cancers that contribute most heavily to worldwide mortality, Ras mutations are extremely common.^3^ Several isoforms of Ras exist, which are implicated in different types of cancer.^2,3^ A member of this family, KRas-4B, is often found in common and life-threatening cancers, such as lung cancer, colon cancer, and pancreatic cancer.^3^

**Figure 1:**
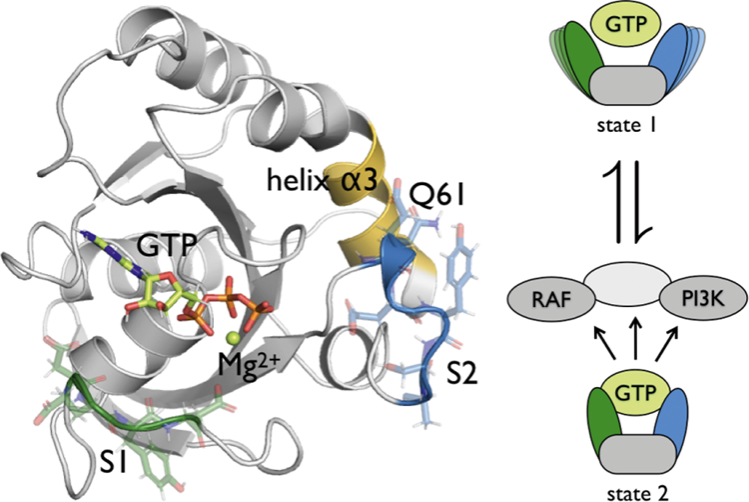
Ras structure and function. Structure of GTP-bound KRas in the active state 2 (left) and a schematic representation of the inactive state 1 and the active state 2 of GTP-bound KRas (right). In the protein drawing, the switch regions are highlighted in green for S1 and blue for S2, helix α3 is highlighted in yellow. The protein is shown as a ribbon with a transparent stick representation for the amino acids in S1 and S2. GTP is shown as solid sticks, with carbon atoms colored in green, oxygen in red, nitrogen in blue, and phosphorus in orange. Mg^2+^ is shown as a green ball. Note that no consensus has been reached yet on the residues ranges that correspond to S1 and S2.^10,11^ We chose to use a narrow definition that corresponds to the residues that are important for the conformational changes in this study, using residues 30–33 for S1 and residues 60–66 for S2. In the schematic drawing, the S1 region is represented in green, the S2 region in blue, and the rest of the protein in gray. State 1 corresponds to the conformational state in which S1 and S2 are more flexible and not bound to GTP. State 2 corresponds to the conformational state in which both S1 and S2 are bound to GTP. State 2 activates downstream effectors like RAF and PI3K by binding them.

Ras proteins consist of a highly conserved catalytic domain called the G domain and a variable C domain which anchors Ras in the membrane. In this work, we focus on the G domain of the KRas-4B isoform, which contains 166 residues and can be considered as the main signaling unit. This domain contains the guanine nucleotide binding site and two regions that can detect the nature of the bound nucleotide, switch 1, S1, and switch 2, S2. These regions, highlighted in green (S1) and blue (S2) in Figure 1(left), are involved in many interactions between Ras and partners. In the GTP-bound state, Ras interacts with downstream effectors such as the Raf and PI3K kinases.^4^ After hydrolysis of GTP, these loop regions adopt a more open conformation^5^ and exhibit more flexibility, causing Ras to lose the ability to bind to downstream effectors. While bound to GTP, Ras exists in a dynamic equilibrium between a weakly populated state 1 and a dominant state 2.^6,7^ Conformational state 1 is more flexible and open than the closed, ordered state 2, as schematically shown in Figure 1 (right). Crystal structures of Ras bound with GTP analogues are typically in the state 2 form of Ras.^4,8^ However, ^31^P NMR studies report that the switch regions can also adopt disordered conformations when bound to GTP, similar to the GDP-bound state.^9^ This work will focus on the transition between the ordered (state 2) and disordered (state 1) states of GTP-bound KRas-4B.

Even though the role of Ras mutations in tumor formation has long been recognized, Ras is considered undruggable.^12,13^ Targeting direct competition with GTP binding is difficult, as Ras has a picomolar affinity for GTP, with micromolar concentrations of GTP in cancer cells. The absence of a hydrophobic pocket for the binding of small molecules complicates the development of allosteric inhibitors of Ras. Obtaining a more detailed understanding of the dynamics underlying the activation of Ras could provide new insights that eventually could lead to new therapeutic leads. However, probing the dynamics of Ras at sufficient resolution in both space and time proves to be very difficult experimentally.^14^

Molecular dynamics (MD) simulations are well suited to obtain high-resolution insights into protein dynamics.^14^ While it is currently possible to run microseconds of straightforward MD, an investigation of the mechanism and kinetic aspects of protein conformational transitions is not feasible. Transitions occurring on microsecond or longer time scales involve high free energy barriers separating stable conformational states. During an MD simulation, most of the time is spent in the stable states, waiting for a barrier crossing, resulting in poor sampling of the transitions. Protein flexibility often involves more than two stable configurations, requiring the sampling of several transitions. The transition path sampling (TPS) algorithm^15^ addresses this timescale problem by focusing the MD simulations on the barrier regions. TPS is a Monte Carlo (MC) simulation in the space of trajectories and collects an ensemble of short reactive trajectories connecting a predefined initial and final state, without prior knowledge of the transition state region. The speed-up gained using TPS and related techniques is tremendous. Assuming a transition rate in the order of 10 μs^–1^, observing a single transition would require on average 10 μs of MD. In contrast, when using TPS, the barrier region is sampled using MD trajectories of only tens of nanoseconds, thus providing a speed-up in the order of several thousand to a million. Even though path sampling methods like TPS were originally developed for two states, they have been extended to be used with multiple stable states.^16^ In this setup, transitions between any two stable states can be sampled, and therefore, it is possible to generate trajectories that connect different pairs of states. The frequency of such switching between transitions depends on the barrier separating different transition channels. Analysis of the switching behavior between these transition channels provides useful insight into the overall dynamics. Note that switching here refers to switching from one transition to another, and not to the regions in the protein that interact with GTP.

It is still an open question how Ras converts from ordered to less ordered conformational states. Ras-activating mutations include substitutions at glutamine (Q) 61,^17^ which affect the conformational equilibrium of Ras. Changing Q61, located in S2, results in reduced GTPase activity in Ras^18^ and an altered conformational space for KRas-4B.^19^ Replacing Q61 by leucine (L) results in an oncogenic mutant.^10^ By changing a hydrophilic glutamine into a hydrophobic leucine,^20^ the hypothesis is that the conformational space of GTP-bound KRas-4B will change, and alter the transition between state 1 and state 2. In particular the effect of mutations on these transitions is unclear. In this work we present multiple state TPS simulations of KRas-4B and the oncogenic Q61L mutant, showing that indeed S2 displays different dynamics for the two systems. In particular, the wild-type (WT) switches frequently from one transition to another, while the Q61L hardly switches at all. Here, we present a way to qualitatively analyze the kinetics of the switching behavior. Closer examination reveals that the WT S2 can reach the flexible open state via a channel that is not accessible for Q61L. Both WT and Q61L can reach the open state by S2 sliding along a slightly hydrophobic pocket of the α3-helix. However, the Q61L mutation prevents direct solvation of S2, which is possible for the WT protein. As a result, the open, inactive state will occur less frequently in Q61L and the protein is more likely to be in an ordered state. Our results show that our methodology is able to map out the dynamics of a Ras protein and can indicate differences in dynamics between a WT protein and an oncogenic mutant. Moreover, the methodology presented here is able to reveal details on the nature of the altered behavior as caused by the mutation. As such, this work is an example of using MSTPS simulations to characterize protein flexibility on time scales of microseconds and longer.

## Methods

### Structure Generation

The initial GTP-bound KRas-4B structure was constructed from the crystal structure of GppNHp bound HRas (PDB-code: 4EFL).^21,22^ This was done by first using homology modeling (MODELLER v9.16),^23^ using sequential alignment to convert HRas to KRas-4B. Then, the GppNHp was manually modified into GTP, by changing the nitrogen into an oxygen and removing the attached hydrogen. Finally, structures of the protein and the GTP were combined into a single file. The initial structure for the mutant (Q61L) was made from this structure by mutating the glutamine (Q) 61 of this final structure into a leucine (L), using MODELLER.^23^

The initial structures were put inside a dodecahedral periodic box with a minimum distance between the structures and the side of the box of 1 nm. This resulted in boxes with volumes of 228.154 and 230.723 nm^3^ for the wild type (WT) and Q61L, respectively. The boxes were filled with TIP3P water.^24^ Fifty-one of the waters were replaced by 30 Na^+^ and 21 Cl^–^ ions to neutralize the systems and achieve a physiological salt concentration of 0.15 M NaCl. This resulted in total system sizes of 22 561 and 22 857 atoms for the WT and Q61L, respectively.

### Molecular Dynamics

#### Procedure

The initial systems were equilibrated in four steps, consisting of energy minimization, an isothermal equilibration, an isothermal–isobaric equilibration and a 1 ns molecular dynamics simulation. The equilibrated structures were used to run four 100 ns molecular dynamics simulations for both WT and Q61L.

#### Settings

In the molecular dynamics simulations, the atomic interactions were described by the AMBER99SB-ILDN^25^ force field, extended with optimized parameters for the triphosphate chain of GTP.^26^ Long-range electrostatic interactions were treated via the particle mesh Ewald method.^27^ The short-range nonbonded interactions (e.g., electrostatics and van der Waals interactions) were cut off at 1.1 nm.

All of the equilibration was performed with GROMACS v.4.6.5.^28^ The leap-frog integrator was used with a time step of 2 fs. Temperature was kept constant at 310 K using the v-rescale thermostat^29^ using two temperature coupling groups: the first group consisted of the protein, GTP and Mg^2+^, while the second group consisted of water, Na^+^, and Cl^–^. The pressure was kept constant using the Parrinello–Rahman barostat^30^ at a pressure of 1 bar. All bond lengths were constrained using the LINCS algorithm.^31^

The 100 ns production runs were performed with OpenMM (7.1.0.dev-5e53567).^32^ The constraints were changed to only affect bonds including a hydrogen atom, using SHAKE,^33^ the integrator was the Velocity Verlet with velocity randomization (VVVR) integrator^34^ from OpenMMTools v.0.14^35^ and the barostat was the Monte Carlo barostat.^36^ The production simulations were run using the CUDA platform of OpenMM on NVIDIA GeForce GTX TITAN X GPUs.

### Collective Variables and Stable States

The long molecular dynamics runs were visually analyzed to identify stable states, using VMD.^37^ Five types of collective variable functions were used to define the stable states, which are described in Table S1 in Appendix A in the Supporting Information.

For both WT and Q61L the relevant collective variables can be found in Appendix A in the Supporting Information in Tables S2 and S3, for S1 and S2, respectively. These collective variables are composed of the collective variable types described in Table S1 in Appendix A in the Supporting Information. The stable states for S1 were S1-D33, S1-30-32, and S1-open for S2 were S2-GTP, S2-α3, and S2-open. The definitions of all stable states can be found in Table S4 in Appendix A in the Supporting Information.

### Transition Path Sampling (TPS)

In the long molecular dynamics simulations, some transitions spontaneously occurred once in several 100 ns simulations. These transitions were used as the starting transition path for TPS.^15,38^ One TPS simulation was performed for S1, starting from the S1-30-32 to S1-open transition. For S2, three TPS simulations were performed, each starting from a different transition. This was done for both WT and Q61L. The initial trajectories were first equilibrated with a TPS simulation until the first decorrelated transition path (a transition path that has no frames in common with the original path) was obtained. This decorrelated path was used as the starting point for the production TPS simulations.

#### Settings

The TPS simulations were performed with OpenPathSampling (0.1.0.dev-c192493).^39,40^ Multiple state TPS (MSTPS)^16^ was performed with an all-to-all flexible length ensemble, excluding self-transitions. All-to-all means all transitions connecting two states are allowed. A self-transition is a path that starts in a state and returns to that same state after crossing the boundaries set by the state definitions. We used the one-way shooting algorithm,^41^ with uniform shooting point selection. For the S1 simulations, 1000 shooting trials were performed, while for each of the S2 simulations, 2000 shooting trials were performed.

#### Analysis

All analysis of the TPS simulations was performed using the tools included in the OpenPathSampling package,^39,40^ extended with custom Python code. Matplotlib^42^ was used for plotting the graphs and triangles.

In Appendix B in the Supporting Information, Figures S1 and S2 show the type of transition as a function of the MC trial for S2 of WT and Q61L.

#### Path Density Histograms

Path density histograms (pdhs) are two-dimensional histograms that show the configurations in a transition path, projected on collective variables. Each path is weighed with its MC weight, and divided by the number of total MC trials. For example, if a trajectory visits a histogram bin, the count of that bin is increased by the MC weight of that trajectory. It does not matter how often the trajectory visits a bin, it counts the trajectory only once. The path density gives the reactive flux of trajectories, whereas regular projection would give a configurational density which is usually overwhelmed by intermediate states.

#### Switching Analysis

**Figure 2:**
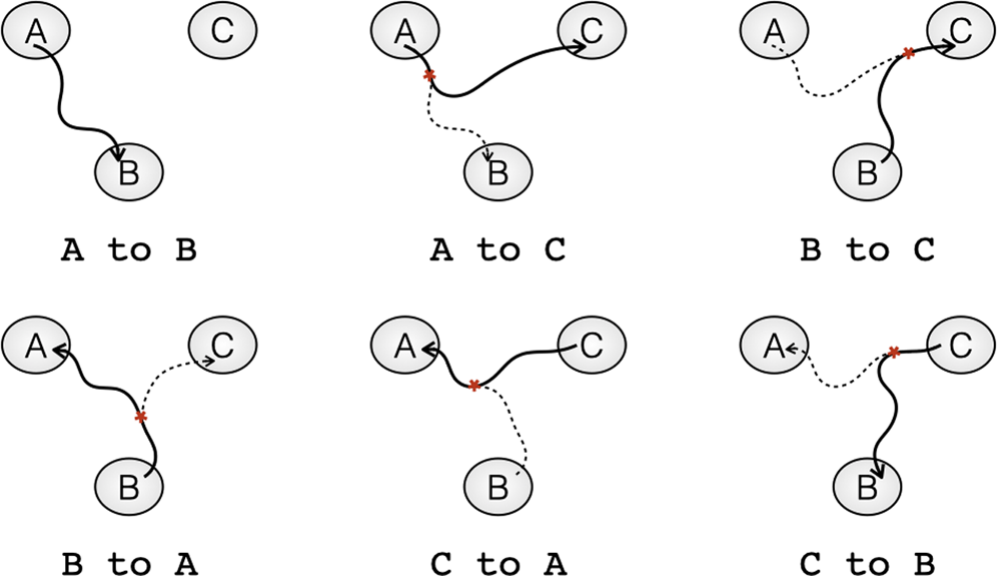
Schematic overview of switching transitions in a three-state system. The three states are labeled A, B, and C. The current path is indicated by a solid line. The previous accepted path is indicated by a dashed line. Shooting points are indicated by an asterisk.

In MSTPS simulations, more than one transition is possible (e.g., A → B, A → C, B → A, etc.); however, only one transition is sampled at a given MC step of the MSTPS simulation. With one-way shooting, an initial A → B trajectory can produce a trial A → C trajectory if a forward trial ends in state C as schematically shown in Figure 2. Such a transition of transitions is called a “switch”. Analyzing the switching behavior provides useful insight into the transition region.

With one-way shooting, switching between a transition A → B and its reversed version, B → A, requires at least three sequential switches: e.g., starting from an A → B transition, a transition from A → C can be generated, followed by a B → C transition, from which the next shot can result in a B → A transition; see Figure 2 for a visualization. One-way shooting can only change the starting or ending state with a backward or forward shot, respectively, but cannot change both in the same MC step. As MSTPS samples an equilibrium distribution, the number of paths collected from the A → B transition should be similar to the number of paths from B → A, reversed in time, which provides a measure for convergence of the simulation.

This measure helps to provide heuristics to assess the convergence of the MSTPS simulations. First, it is an estimate of the ergodicity of the simulation whether all transitions are visited. Second, forward and backward versions of transitions with the same pair of states (e.g., A → B and B → A) should have similar statistics in all ways. Furthermore, the fraction of MC steps spent in the two transitions between the same pair of states should be the same. The same goes for the path length distributions.

#### Kinetics Analysis

As we assume that the switching samples an equilibrium distribution, the probability *P*_*i*_ of sampling a transition *i* is given by
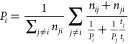
1where *n*_*ij*_ is the number of switches from *i* to *j* and *t*_*i*_ is the number of MC steps sampling transition *i*. As the sum of all probabilities is equal to 1, ∑_*i*_*P*_*i*_ = 1, eq 1 can be solved for all *P*_*i*_. From the probabilities, the switching rate from *i* to *j*, *k*_*ij*_, can be calculated by
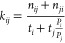
2

The values for *n* and *t* are taken from the MSTPS simulations. This analysis is adapted from ref (24).^43^

## Results and Discussion

### Identification of Conformational States

**Figure 3:**
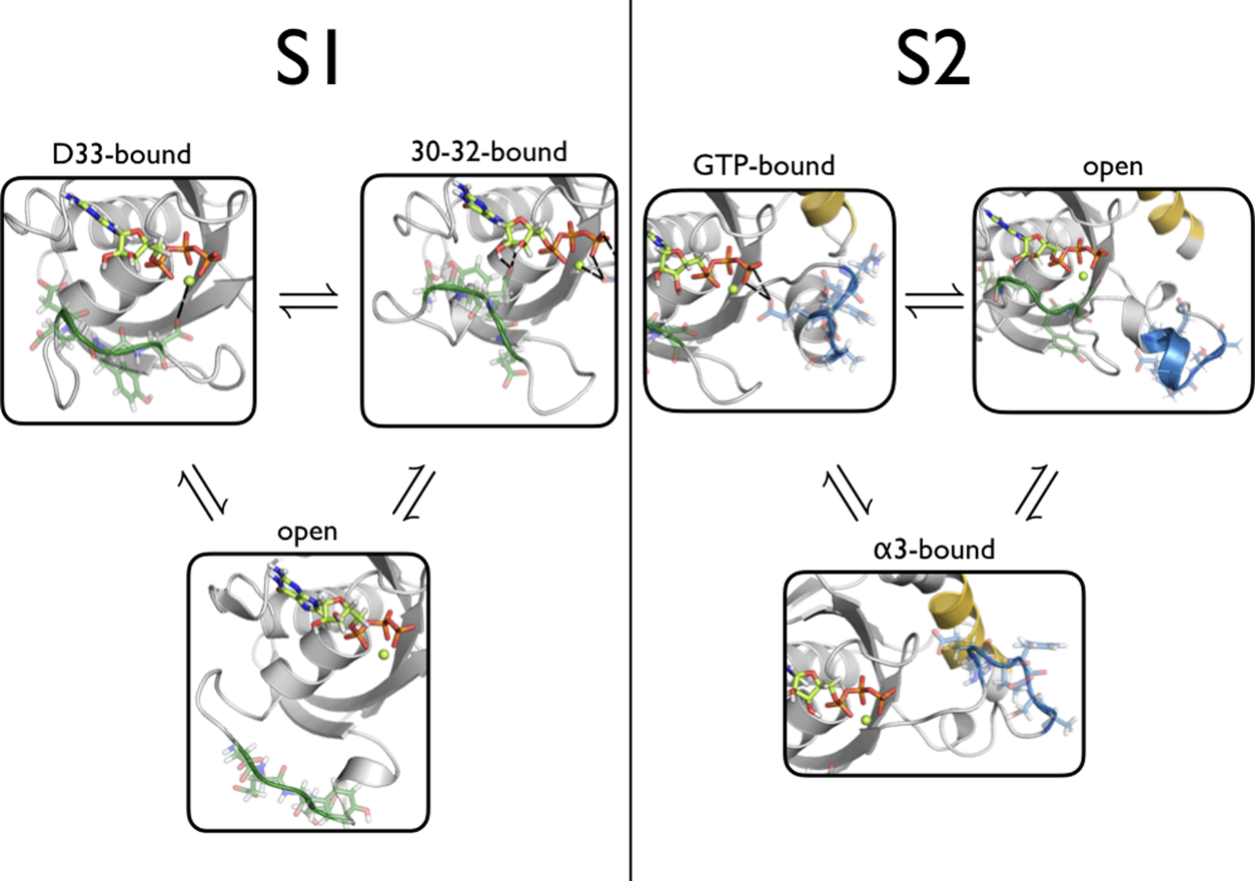
Stable states of KRas. The stable states found for S1 and S2 are shown with the same coloring as Figure 1 (left). The S1-D33 state corresponds to the conformation in which D33 in S1 has a hydrogen bond with GTP. The S1-30-32 state corresponds to the conformation in which one or more hydrogen bonds occur between residues 30–32 and GTP. The S1-open state corresponds to the conformation in which S1 has no interactions with GTP and is oriented away from GTP. For S2, the S2-GTP state corresponds to the conformation in which S2 has one or more hydrogen bonds with GTP. The S2-α3 state corresponds to a state in which S2 has no interactions with GTP and is oriented away from GTP. The S2-α3 state corresponds to a conformation in which S2 has no interactions with GTP, but instead has four interactions with the α3-helix.

The crystal structure of GppNHp bound HRas (PDB: 4EFL)^21,22^ was used as a structural template to model the sequence of WT and Q61L KRas-4B with GTP bound. With these two structures we performed four 100 ns MD simulations to explore the conformational space of KRas, for both WT and Q61L. These simulations resulted in the characterization of two stable states for S1, S1-D33 and S1-open and two stable states for S2, S2-GTP and S2-open. After initial TPS simulation ended up with trajectories that did not end in any of these states, a third state was found for S1, S1-30-32, as well as S2, S2-α3. All of the stable states are shown in Figure 3. When S1 is in the S1-D33 state, the side chain of D33 is involved in (water-mediated) hydrogen bond interactions with GTP. For the S1-30-32 state, S1 has shifted along GTP, compared to the S1-D33 state, to form one or more hydrogen bonds between the side chains of residues D30, E31, or Y32 and GTP. The conformations in which S1 has no hydrogen bond interaction with GTP and where it is oriented away from GTP are classified as the S1-open state. The S2-GTP state corresponds to the conformation of S2 where it forms hydrogen bonds with GTP. Two states can occur when S2 is oriented away from GTP. In the S2-α3 state, S2 has multiple interactions between its side chains and the α3-helix. In the S2-open state, S2 has no binding interactions with GTP. The parameters for defining these states are listed in Appendix A. Within the timescale of the path sampling simulations, the conformation of S1 has little effect on the conformation of S2 or vice versa. The same stable states were found for both WT and Q61L and were stable for at least 50 ns of MD. Note that no consensus has been reached yet on the range of residues that correspond to each switch region.^10,11^ We chose to use a narrow definition that includes the residues that are important for the conformational changes in this study, using residues 30–33 for S1 and residues 60–66 for S2. Adding more residues will not change the stable state definitions.

### Mapping Conformational Transitions

Using MSTPS, we investigated the transitions between the stable states as identified in the MD simulations for S1 and S2 separately. For both S1 and S2, three pairs of transitions are observed: S1-D33 ↔ S1-30-32, S1-D33 ↔ S1-open, and S1-30-32 ↔ S1-open for S1, and S2-GTP ↔ S2-α3, S2-GTP ↔ S2-open, and S2-α3 ↔ S2-open for S2. For both WT and Q61L we performed one MSTPS simulation for S1 starting at the S1-30-32 ↔ S1-open transition, and three independent MSTPS simulations for S2, each starting in a different transition, resulting in eight MSTPS simulations in total. We decided to focus mainly on S2, as this region contains the mutation. The statistics of the MSTPS simulations are listed in Table 1 and indicate a good acceptance ratio of 33% or higher, and an aggregate simulation time of microseconds.

**Table 1:** Statistics of the MSTPS Simulations^a^

	S1 WT	S1 Q61L	S2 WT			S2 Q61L		
	sim 1	sim 1	sim 1	sim 2	sim 3	sim 1	sim 2	sim 3
MC steps	1000	1000	2000	2000	2000	2000	2000	2000
accepted steps	355	334	766	824	787	688	748	759
acceptance	35.5%	33.4%	38.3%	41.2%	39.4%	34.4%	37.4%	38.0%
decorrelated trajectories	50	57	131	151	128	99	129	125
average path length (ns)	5.94	2.28	3.49	1.26	1.50	1.42	1.36	1.98
total simulation time (μs)	4.88	2.67	6.01	2.15	2.10	2.26	1.83	3.74

a MC steps indicates the number of Monte Carlo trials, also called shooting moves. Accepted steps refers to the number of MC trials that were accepted, and the acceptance is . Decorrelated trajectories indicate the number of accepted trajectories that do not have any frames in common. The total simulation time is the total time of MD performed by the MD engine in the MSTPS simulations.

Path sampling simulations for proteins (with stochastic dynamics and diffuse barriers) commonly employ the stochastic, or “one-way” shooting algorithm,^38^ which improves the acceptance ratio. In this algorithm, a trial move replaces only part of the trajectory (forward or backward). Therefore, successive trajectories will have segments with overlapping frames and at least two trials (one forward and one backward) are needed for an accepted trajectory to have no frames in common with the original. These no-overlap trajectories are referred to as “decorrelated” and are required for sufficient sampling. Table 1 shows that each simulation generated on average 100 decorrelated trajectories.

**Figure 4:**
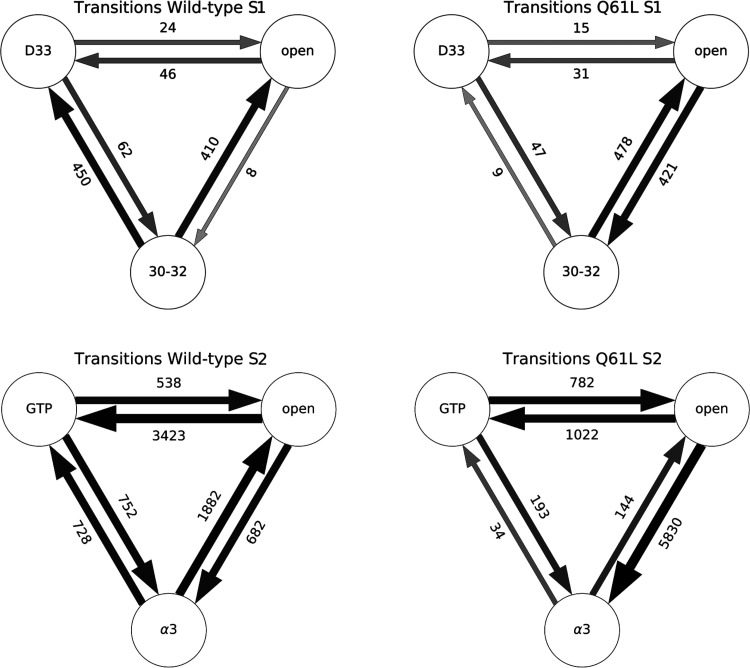
Transitions of S1 and S2 in WT and Q61L. Schematic representation of the number of accepted paths per transition for S1 (top) and S2 (bottom) for the WT (left) and Q61L (right) TPS simulations. The circles correspond to the stable states. For S1, D33 indicates the S1-D33 state, 30–32 the S1-30-32 state, and open the S1-open state. For the S2 plots, GTP is the S2-GTP state, open the S2-open state, and α3 the S2-α3 state. Arrows represent the sampled transition, pointing in the direction that was considered forward during the simulation. The labels are the number of accepted paths in each transition, and the width and the color of the arrows are scaled with a 10 log scale of this number.

The transitions sampled in the MSTPS simulations are summarized in Figure 4. Between each pair of states, there are two possible transitions, corresponding to what is the forward time direction in the path. Although MSTPS only samples one transition at a time, switching between transitions can occur when there are more than two states. For example, given states A, B, and C, an initial A → B trajectory can produce a trial A → C trajectory if a forward trial ends in state C. Such a transition in transitions is called a “switch”. Note, this switch between transitions is not to be confused with the regions in KRas called switch 1 and switch 2, which we abbreviated as S1 and S2, respectively. The Methods section contains a more detailed explanation of transition switching with examples. Analyzing the switching behavior provides useful insight into the transition region. A lack of switching between two states indicates there is a large (free energy) barrier in the transition region between the channels for the individual reactions. Conversely, many switching events suggests a flatter, more diffusive landscape in the transition region. Both the WT and the Q61L simulations have sampled all transitions for both S1 and S2.

At infinite sampling, the relative sampling frequency for the two transitions between a given pair of states will be identical. Most transitions in all four simulation setups show that the number of samples in both directions of each transition is within the same order of magnitude, except for the 30-32 ↔ D33 and the 30-32 ↔ open in the S1 WT simulation, and the S2-open and S2-α3 pair in the S2 Q61L simulation. In these cases, switching to another transition has become a rare event.

**Figure 5:**
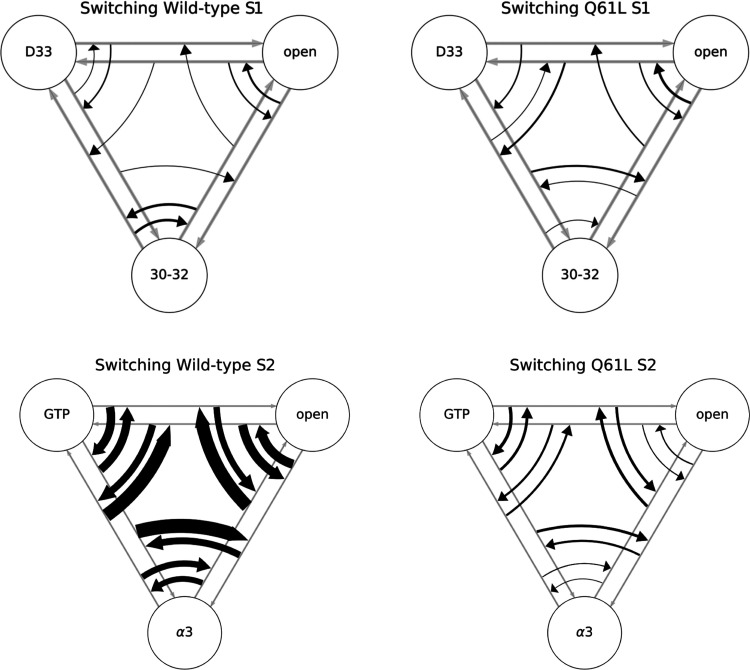
Switches between transitions. Schematic representation of the amount of switching between different sampled transitions in WT (left) and Q61L (right), for both S1 (top) and S2 (bottom). Circles represent the stable states, and gray arrows show the unscaled transitions. The same state abbreviations were used as in Figure 4. Each of the black arrows represents a switch between the transitions, scaled linearly to the number of times this switch occurred.

To obtain more insight into the sampling, the Supporting Information contains plots of the transition sampled in each accepted path as a function of the number of MC steps. Figure S6 in Appendix D in the Supporting Information shows the transition per accepted path for the S1 simulations and Figures S1 and S2 in Appendix B in the Supporting Information show these data for the S2 simulations. The data in these figures are summarized in Figure 5, which shows the number of switches between the six transitions occurring for S1 and the six transitions of S2 as arrows with a thickness relative to the number of switches. The switching looks very similar between WT and Q61L for S1; however, this is not the case for S2. Clearly, the number of switches between transitions in S2 is much lower for the mutant than for the WT, indicating that the WT has a lower free energy barrier between the different transition channels than Q61L. The difference in the sampling of the S2-open and S2-α3 transition in WT and Q61L suggests that the mutation has altered the transition region. Such an alteration can happen in two ways, that do not exclude each other. The mutation changes the stability of one or more of the stable states, thus making transitions less (or more) likely. The mutation can also alter the mechanism of the transition, which has a direct effect on the transition region.

Representative trajectories of all sampled transitions of both the WT and the Q61L were visually compared. No distinct differences in transition mechanisms between WT and the Q61L were observed. In conclusion, these results suggest that the mutation in S2 has little effect on the dynamical behavior of S1. Further analysis is done only on S2 in the following sections, as the S2 data showed the largest difference.

### Kinetic Analysis of Switching of S2

**Figure 6:**
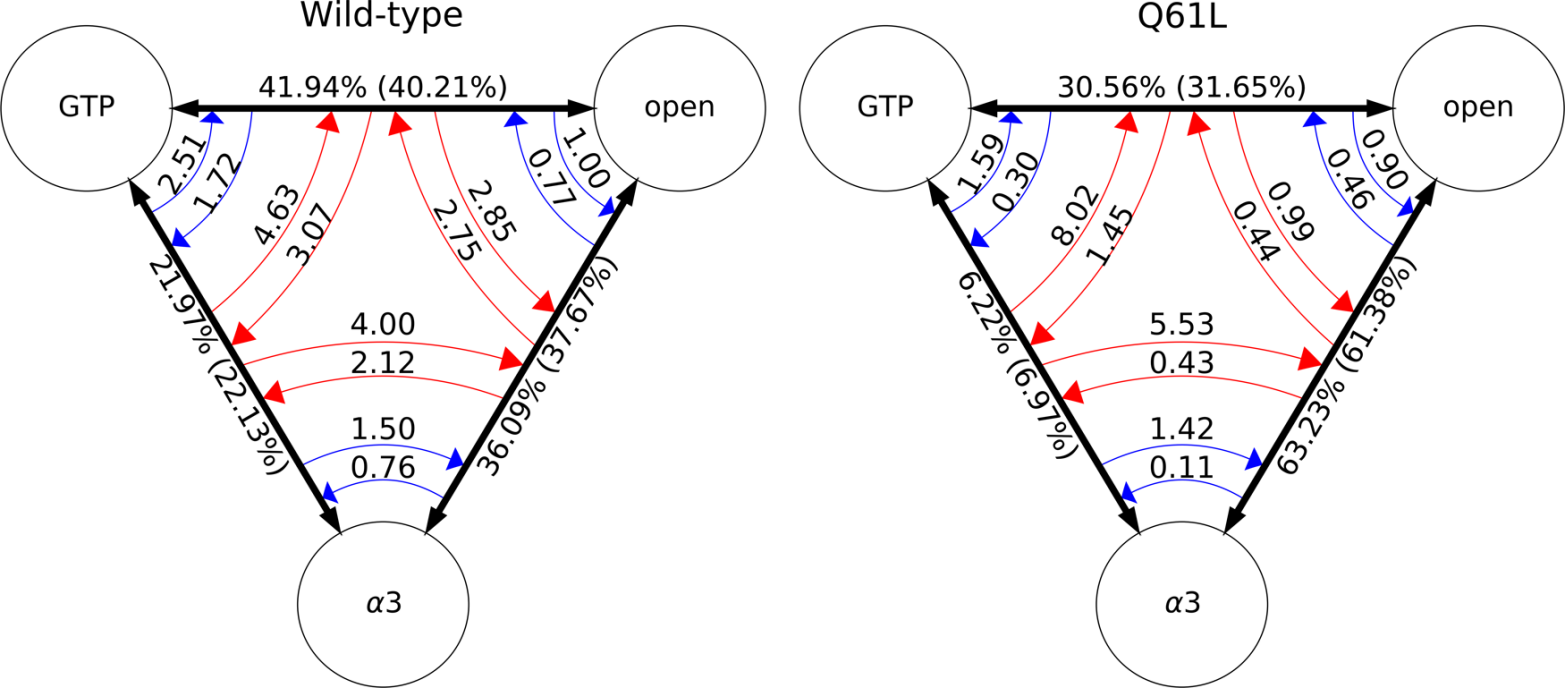
Population analysis. (Left) All WT S2 simulations and (right) Q61L simulations starting in the S2-α3 → S2-GTP transition and the S2-open → S2-α3 transition. Circles represent the stable states, and the same state abbreviations are used as in Figure 4. Black arrows are the time combined transitions, red arrows are the switching rates obtained from all accepted paths, and blue arrows are the switching rates observed from only using decorrelated paths. The labels of the transitions arrows are the population percentages from all accepted paths, with the decorrelated data in parentheses. The labels of the switching arrows are the rates of switching per 100 MC steps.

To quantify the relative frequency or “population” of each transition and the switching rate between transitions, we applied a kinetics analysis approach as developed for Replica Exchange MD^43^ on our MSTPS data. In this analysis, rate constants are estimated from the number of transitions between stable states and the average residence times in the stable states. We performed an analogous analysis on the MSTPS data, with the following definitions: time corresponds to the MC trials, stable states correspond to transitions between states and transitions correspond to switches, i.e., transitions of transitions. Such a kinetics analysis results in rate constants that measure the switching rate between transitions in units of MC steps, see the Methods section for more detail. We analyzed the kinetics by including all accepted trajectories, or only decorrelated trajectories; see Figure 6. For one Q61L simulation, starting from S2-GTP → S2-open, no switching occurs out of the S2-GTP ↔ S2-α3 transition and we therefore excluded this simulation from the switching analysis. The “population” of the S2-α3 ↔ S2-open transition is almost twice as high for Q61L (63.23%) than for WT (36.09%), while the S2-GTP ↔ S2-α3 is less likely for Q61L (6.22%) than for WT (21.97%).

When including all accepted paths, most switching rates are lower for Q61L than for WT, except for the switches out of the S2-GTP ↔ S2-α3 transition.

The largest relative difference between WT and Q61L is observed for the switching rates out of the S2-α3 ↔ S2-open transition. When including only decorrelated trajectories, all switching rates are lower for Q61L, with the largest relative difference for the transition into the S2-GTP ↔ S2-α3 transition. These observations indicate that the barrier separating S2-α3 and S2-open states is lower in Q61L than in WT. In addition, it is difficult to obtain decorrelated paths for the S2-GTP ↔ S2-α3 transition. This is also apparent from Figures S1 and S2 in Appendix B in the Supporting Information, where the residence time in the S2-GTP ↔ S2-α3 transitions is almost always only a single MC step. Less switching would occur if the transition channels in the free energy landscape are less deep. An alternative view is that part of the region between transitions would be less favorable for Q61L compared to WT. Inspection of the least changed path, a trajectory connecting paths on top of the transition barrier, in Figures S3 and S4 in Appendix C in the Supporting Information, confirms that the observed switching is indeed a diffusive process and that the Q61L mutation constrains the dynamics.

### Path Densities Reveal Two Channels for S2

**Figure 7:**
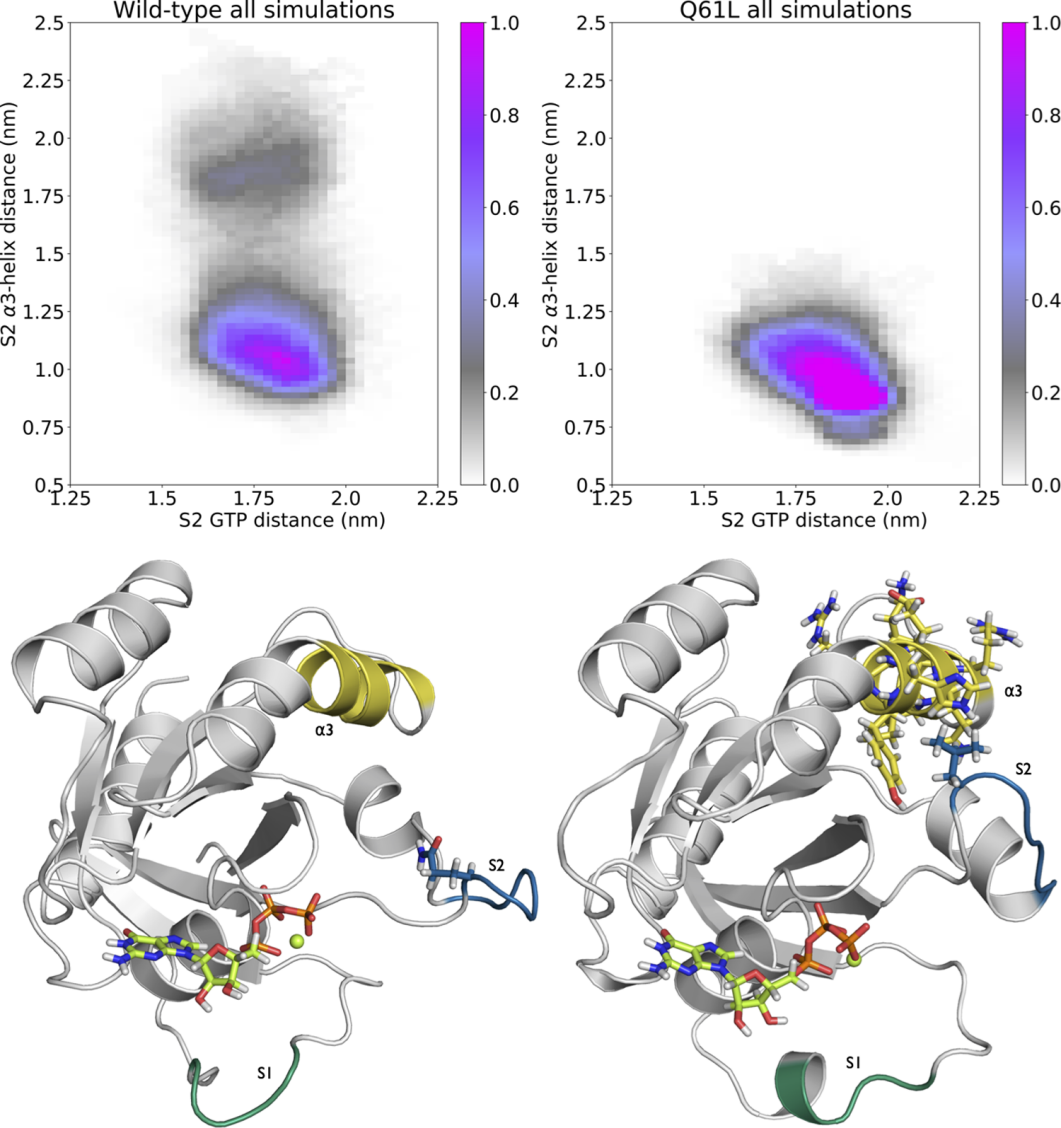
Mechanistic differences between WT and Q61L. (Top) Path density histograms of the WT (left) and Q61L (right) simulations with the distance between the circular mean center of mass (cCOM) of S2 and the cCOM of GTP on the *x*-axis and the distance between the cCOM of S2 and the cCOM of residues 95, 96, 99, and 102 on the *y*-axis. The bin widths are 0.25 Å for both axes. The coloring shows the sampled configuration of the transitions, weighted per trajectory, and normalized to 1. The stable states are not defined entirely based on these coordinates, but S2-GTP corresponds roughly to the area left of 1.6 nm on the *x*-axis, S2-open to the area right of 2 nm on the *x*-axis, and S2-α3 at intermediate distances on the *x*-axis, and below 0.8 on the *y*-axis. (Bottom) Snapshots of the state with the same coloring as Figure 1 (left) from (left) the channel that is far away from the α3-helix in the WT simulation and (right) the channel that is close to the α3-helix in the Q61L simulation.

To further investigate the origin of the difference in switching kinetics, we projected the trajectory space sampled in the combined TPS simulations in a path density histogram. Such histograms show the reactive flux of trajectories projected onto collective variables. Figure 7(top) shows the path density for the WT (left) and Q61L (right) S2 TPS simulations, projected in the plane of the distances between S2 and GTP, and between S2 and residues H95, Y96, Q99, and R102 of the α3-helix (see Table S3 in Appendix A in the Supporting Information for definitions of these distances). Note that path densities do not show stable states, as the trajectories are stopped when reaching one. Comparing the two path density plots indicate that the WT simulations sample a larger region on the vertical axis, as the WT path density extends to above 1.25 nm, while the Q61L path density is more confined to the region below 1.25 nm in S2-α3 distance. This indicates that S2 can move away from the α3-helix more easily in the WT protein. The WT histogram even shows a second channel for transitions between the S2-GTP and the S2-open states, at a distance of more than 1.75 nm from the α3-helix. The Q61L simulations sample configurations closer to the α3-helix, as indicated by the density below 0.8 nm on the vertical axis. A more pronounced negative correlation exists between the S2-α3-helix and the S2-GTP distances. The further away S2 is from GTP, the closer it is to α3. Furthermore, the Q61L simulations do not sample the second channel at all. As three independent simulations were performed for both WT and Q61L, each initiated from a different transition, the absence of direct solvation transitions for Q61L are likely to be a direct consequence of the mutation.

**Figure 8:**
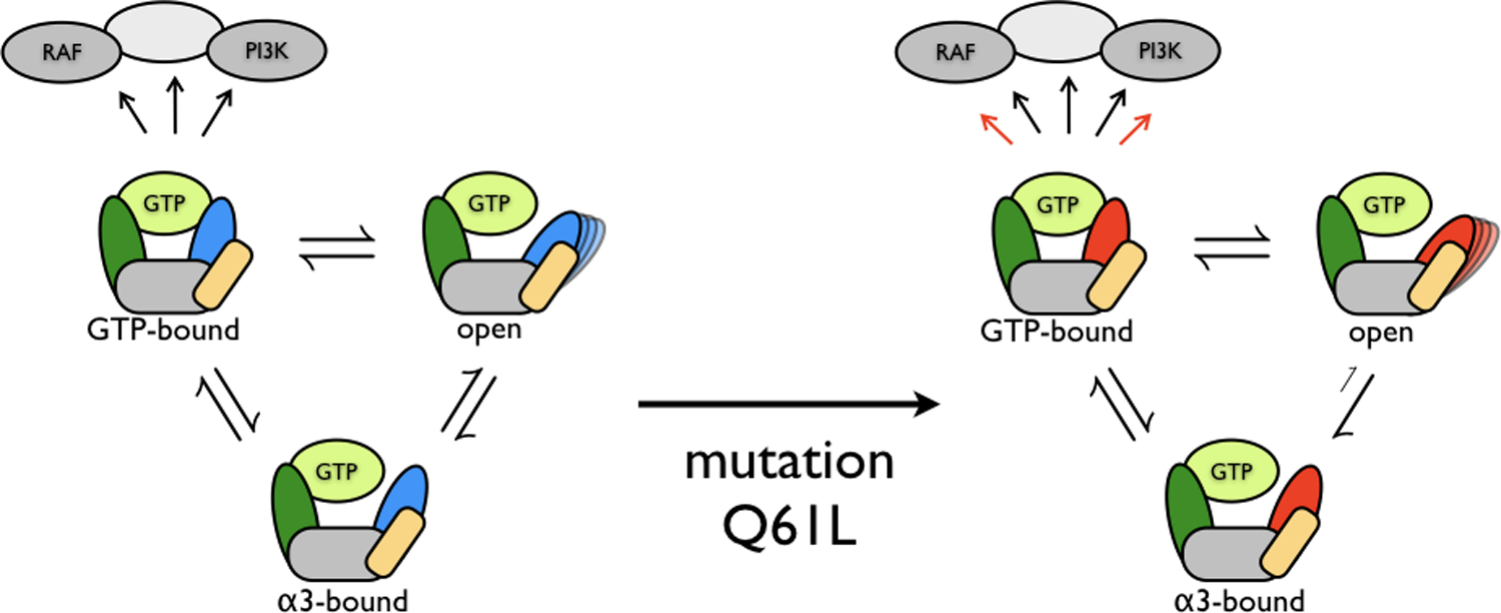
Schematic overview of the effect of the Q61L mutation on the dynamics of KRas. (Left) WT, (right) Q61L. The S1 region is represented in green, the S2 region in blue for WT and red for Q61L, the α3-helix in yellow, and the rest of the protein in gray. Downstream effectors are also shown in gray. Assuming only the S2-GTP-bound state triggers the downstream effectors, the Q61L mutation alters the conformational space such that one channel to reach the open state becomes very unlikely. This would lead to either a shift in the equilibrium distribution between the open and GTP-bound state or to transitions occurring more frequently. Both of these effects would lead to an increased probability to encounter downstream effectors while in the GTP-bound state, which would trigger the downstream signaling networks.

The two conformations plotted in Figure 7(bottom) illustrate the difference between the two reaction mechanisms or channels. The image on the left shows the WT protein in the S2-open state with S2 at a distance of at least 1.75 nm from the α3-helix. On the right, Q61L is shown in the S2-open state with S2 closer than 1.25 nm to the α3-helix. The distance of S2 to α3 is indicative of the different mechanisms. The channel far away from the α3-helix represents a mechanism involving water molecules solvating S2, resulting in S2 extending into the solvent, away from both GTP and the α3-helix. The channel close to the α3-helix represents S2 moving along a hydrophobic pocket on the α3-helix. In this reaction mechanism, S2 can either enter the S2-α3 state by forming four contacts between S2 and the α3-helix, or by sliding along the helix until entering S2-open. The Q61L mutation changes a hydrophilic residue to a hydrophobic one, thus lowering the affinity of S2 for water. Therefore, the solvated transition channel, which is easily accessible for the WT protein, becomes much less likely for Q61L. Moreover, the mutated S2 has stronger interactions with the α3-helix, as shown by the higher path density in the channel close to α3-helix (Figure 7(top, right)), indicating that for Q61L it is harder to escape from the α3-state. The increased stability of the α3-state renders the S2-GTP ↔ S2 transition less likely. Figure 8 summarizes this conclusion.

### Q61L Has a Higher Propensity for a More Structured S2-Open

Visual inspection of the transition paths shows that in some WT trajectories the α2-helix (residues 65–73, overlapping with part of S2), unfolds when entering the S2-open state, but retains its shape for the Q61L mutant. Probability histograms of the S2-open state obtained from the transition path ensemble by projection on the number of helical hydrogen bonds in the α2-helix and the S2-α3-helix distance shown in Figure S5 in Appendix C in the Supportin Information further substantiate this observation. Therefore, we can conclude that the S2-open state contains multiple substates, characterized by the conformation of the α2-helix and the S2-α3 distance. Furthermore, these probability histograms show that Q61L has a higher propensity compared to WT for the more structured conformations of the S2-open state. The α2-helix plays a vital role in binding other proteins,^5^ suggesting that these structured substates are more similar to the active state 2 than the inactive state 1.

The more open and flexible substates of the S2-open state are less likely to be recognized by downstream effectors. A β-strand in the PI3 kinase interacts with KRas via both S1 and S2,^4^ which can only occur when both S1 and S2 are in a closed conformation. The more open conformations are harder to reach in Q61L, and indeed, we only observe these flexible substates in the WT simulations, thus providing an explanation for the increased probability of Q61L to bind a downstream effector. This prediction may be tested by repeating the NMR experiment as performed by Geyer et al.,^9^ comparing the effect of the Q61L mutation on the switching frequency. Alternatively, the lifetime of the S2-α3 state could be measured using ^15^N NMR spectroscopy, by labeling nitrogen atom NE2 in the Q95 side chain, located in the α3-helix. Finally, the α3-helix identified as important for the transitions between the ordered, active state 2 and the flexible, inactive state 1 might provide a new target for the development of compounds that could ameliorate the effect induced by the Q61L mutation.

## Conclusions

In this work, we investigated the conformational space and dynamic behavior of KRas in complex with GTP using multiple state transition path sampling. The loops S1 and S2 in KRas that interact with GTP each visit three different conformational states. Surprisingly, these conformational states do not change upon introducing the Q61L mutation, located in region S2. However, the mutation has a significant effect on the transitions between the conformational states of region S2. This effect could be a result of changes in the relative free energies of the conformational states or changes in the transition mechanisms, or a combination of both. While the WT protein frequently changes from one transition to another, the mutant hardly changes at all. A closer examination of the various transitions revealed that S2 in the WT protein is more likely to be solvated than in the Q61L mutant. The Q61L mutation prevents direct solvation of S2, which is an accessible route for the WT protein. Both WT and mutant can reach the opened-up state by S2 sliding along a slightly hydrophobic pocket on the α3-helix.

Our results show that the MSTPS methodology in combination with the switching analysis is able to map out the dynamics of a Ras protein, indicate differences in dynamics between the WT protein and an oncogenic mutant, and reveal details on the nature of the altered behavior as caused by the mutation. As such, this work is an example of a detailed characterization of protein flexibility, and the effect of point mutations on this flexibility. The predictions from our work can be tested experimentally using techniques based on NMR or infrared spectroscopy and may provide a new target for the development of compounds that could diminish the effect of the Q61L mutation.

## Supporting Information Available

Stable state definitions (Appendix A); transitions as a function of the Monte Carlo steps (Appendix B); additional MSTPS results for S2 (Appendix C); and MSTPS results for S1 (Appendix D) (PDF)
