# Supplementary material for: Path Sampling Simulations Reveal How the Q61L Mutation Alters the Dynamics of KRas
Source: J Phys Chem B. 2022 Nov 25;126(48):10034–44. doi: 10.1021/acs.jpcb.2c06235 (PMC9743084; doi:10.1021/acs.jpcb.2c06235)
Supplement: Supplementary file 1 — jp2c06235_si_001.pdf [file jp2c06235_si_001.pdf]

Supporting information for Path sampling simulations reveal how the Q61L mutation alters the dynamics of KRas

Sander Roet,[†,‡] Ferry Hooft,[†] Peter G. Bolhuis,[†] David W.H. Swenson,[†,¶,§] and Jocelyne Vreede[*,†]

†*van 't Hoff Institute for Molecular Sciences, University of Amsterdam, Science Park 904, 1098 XH, Amsterdam, The Netherlands*

‡*Department of Chemistry, Norwegian University of Science and Technology (NTNU), NO-7491, Trondheim, Norway*

¶*Univ Lyon, ENS de Lyon, Univ Claude Bernard, CNRS, Laboratoire de Physique and Centre Blaise Pascal, 69007 Lyon, France*

§*Current address: Open Free Energy; Open Molecular Software Foundation; Davis, CA 95618*

E-mail: J.Vreede@uva.nl

Supporting Information Available

Appendix A: Stable state definitions The stable states are defined by ranges in collective variables. This appendix provides a guide to these stable state definitions. Table 1 gives the types of collective variables, while Tables 2 and 3 list the collective variables used to define the stable states for S1 and S2, respectively. Table 4 gives the ranges in collective variable space for the stable states found for S1 and S2.

Table 1: **List of the different collective variable types.**

CV type	Description
Minimum distance	The smallest distance between two groups of atoms. Using MDTraj,[1] distances of every atom pair were calculated. The lowest is the minimum distance.
Circular mean center of mass (cCOM)*	The circular mean center of mass (cCOM) is a center of geometry calculation that allows for periodicity. The system is first mapped onto a cube, followed by the calculation of the center of mass, using the procedure of ref.[2] Then the cCOM is mapped back onto the original axes.
Number of hydrogen bonds	The number of hydrogen bonds is calculated by counting how many of the possible donor-acceptor pairs form a hydrogen bond. A hydrogen bond in this code is defined by having a H_{donor}-acceptor distance smaller than 0.25 nm and having an X_{donor}-H_{donor}-acceptor angle larger than $\frac{2}{3}\pi$ rad.
Number of water mediated hydrogen bonds	The number of water mediated hydrogen bonds is calculated by first selecting all water oxygens that are within a distance of 0.35 nm from both input groups. If a water forms a hydrogen bond with both groups, it is counted as a water mediated hydrogen bond. The hydrogen bond calculation is done as described above.
Number of bonds	The number of bonds is the number of pairs, from a given list of pairs, for which the minimum distance is smaller than 0.35 nm. The minimum distance is defined in the minimum distance cv type.

* Note that this circular mean center of mass does not give the actual center of mass.

In the CV type column the name of the collective variable type as used in table 2 and 3 are shown, with their description in the Description column.

Table 2: **List of the relevant collective variables for the stable state definitions of S1.**

CV	Description
d_GTP_asp30[a]	The minimum distance between the C_γ of aspartic acid 30 and the heavy atoms of GTP, including Mg^{2+}.
d_GTP_glu31[a]	The minimum distance between the C_δ of glutamic acid 31 and the heavy atoms of GTP, including Mg^{2+}.
d_GTP_tyr32[a]	The minimum distance between the side-chain oxygen of tyrosine 32 and the heavy atoms of GTP, including Mg^{2+}.
d_GTP_asp33[a]	The minimum distance between the C_γ of aspartic acid 33 and the heavy atoms of GTP, including Mg^{2+}.
n_hbonds_GTP_asp30[c]	The number of hydrogen bonds between the side-chain oxygens of aspartic acid 30 and the hydroxyl groups on the ribose of GTP.
n_hbonds_GTP_tyr32[c]	The number of hydrogen bonds between the hydroxyl oxygen of tyrosine 32 and the hydroxyl groups on the ribose of GTP.
n_hbonds_tyr32_GTP[c]	The number of hydrogen bonds between the oxygens of GTP and the hydroxyl group of tyrosine 32.
n_hbonds_ile55_tyr40[c]	The number of hydrogen bonds between the backbone carbonyl of isoleucine 55 and the backbone amide of tyrosine 40.
n_hbonds_GTP_S1[c]	The number of hydrogen bonds between the backbone carbonyls of valine 29 and aspartic acid 30 and the hydroxyls on the ribose of GTP.
n_h_med_bonds_GTP_asp33[d]	The number of water mediated hydrogen bonds between all oxygens of aspartic acid 33 and all oxygens of GTP.
n_h_med_bonds_MG_asp33[d]	The number of water mediated hydrogen bonds between all oxygens of aspartic acid 33 and Mg^{2+}.
n_h_med_bonds_GTP_nnb[d]	The number of water mediated hydrogen bonds between the side-chain oxygens of aspartic acid 30, glutamic acid 31 and tyrosine 32 and all oxygens of GTP.
n_h_med_bonds_MG_nnb[d]	The number of water mediated hydrogen bonds between the side-chain oxygens of aspartic acid 30, glutamic acid 31 and tyrosine 32 and Mg^{2+}.

[a] This collective variable uses the minimum distance as described in table 1.

[c] This collective variable uses the number of hydrogen bonds as described in table 1.

[d] This collective variable uses the number of water mediated hydrogen bonds as described in table 1.

Table 3: **List of the relevant collective variables for the stable state definitions of S2**

CV	Description
d_gly12_gly60[a]	The minimum distance between the heavy atoms of glycine 12 and the heavy atoms of glycine 60.
d_gly12_gln61[a,wt]	The minimum distance between the heavy atoms of glycine 12 and the side-chain heavy atoms of glutamine 61.
d_gly12_leu61[a,Q61L]	The minimum distance between the heavy atoms of glycine 12 and the side-chain heavy atoms of leucine 61.
d_GTP_glu62[a]	The minimum distance between the C_δ of glutamic acid 62 and the heavy atoms of GTP, including Mg^{2+}.
d_GTP_glu63[a]	The minimum distance between the C_δ of glutamic acid 63 and the heavy atoms of GTP, including Mg^{2+}.
d_cCOM_GTP_S2[a,b]	The minimum distance between the circular mean center of mass of all atoms of residues 61 to 66 and the circular mean center of mass of all atoms of GTP, including Mg^{2+}.
n_S2_α3[e]	The number of combinations between the sets of {histidine 95, tyrosine 96, glutamine 99, arginine 102} and {{61}*, glutamic acid 62, glutamic acid 63, tyrosine 64} for which the minimal distance between the side-chain heavy atoms of the residue from the first set and all heavy atoms of the residue from the second set is smaller than 0.35 nm.

[a] This collective variable uses the minimum distance as described in table 1.
[b] This collective variable uses the circular mean center of mass as described in table 1.
[e] This collective variable uses the number of bonds as described in table 1.
[wt] Only used in wild-type KRas.
[Q61L] Only used in the Q61L mutant of KRas.
* {61} is glutamine 61 for wild-type KRas and leucine 61 for the Q61L mutant of KRas.

These definitions apply to both WT and Q61L. The CV column shows the collective variable names, with their description in the Description column.

Table 4: **List of the stable state definitions for KRas.**

State	CV	Constraint(s)	Logic
S1-D33	d_GTP_asp33	$0.0 \leq x \leq 0.43$ nm	
	n_h_med_bonds_GTP_asp33	$1.9 \leq x \leq 5.0$ bonds	*or*[a]
	n_h_med_bonds_MG_asp33	$0.9 \leq x \leq 5.0$ bonds	
S1-30-32	d_GTP_asp30	$0.0 \leq x \leq 0.35$ nm	*and*[b] (d_GTP_asp30, n_hbonds_GTP_asp30)
	n_hbonds_GTP_asp30	$1.9 \leq x \leq 5.0$ bonds	
	d_GTP_glu31	$0.0 \leq x \leq 0.42$ nm	
	n_hbonds_GTP_tyr32	$0.9 \leq x \leq 4.0$ bonds	*or*[a] (from the *and* group through n_h_med_bonds_MG_nnbound)
	n_hbonds_tyr32_GTP	$0.9 \leq x \leq 4.0$ bonds	
	n_h_med_bonds_GTP_nnbound	$1.9 \leq x \leq 5.0$ bonds	*and*[b] (the *or* group with d_GTP_asp33)
	n_h_med_bonds_MG_nnbound	$0.9 \leq x \leq 5.0$ bonds	
	d_GTP_asp33	$1.1 \leq x \leq 5.0$ nm	
S1-open	d_GTP_asp30	$1.05 \leq x \leq 5.0$ nm	
	d_GTP_glu31	$1.55 \leq x \leq 5.0$ nm	
	d_GTP_tyr32	$1.25 \leq x \leq 5.0$ nm	*and*[b]
	d_GTP_asp33	$1.25 \leq x \leq 5.0$ nm	
	n_hbonds_ile55_tyr40	$-0.1 \leq x \leq 0.1$ bonds	
	n_hbonds_GTP_S1	$-0.1 \leq x \leq 0.1$ bonds	
S2-GTP	d_gly12_gly60	$0.0 \leq x \leq 0.3$ nm	
	d_gly12_{61}*	$0.0 \leq x \leq 0.3$ nm	*or*[a] (first four rows)
	d_GTP_glu62	$0.0 \leq x \leq 0.65$ nm	
	d_GTP_glu63	$0.0 \leq x \leq 0.65$ nm	*and*[b] (the *or* group with d_cCOM_GTP_S2)
	d_cCOM_GTP_S2	$0.0 \leq x \leq 1.6$ nm	
S2-α3	n_S2_α3	$3.5 \leq x \leq 4.5$ bonds	*and*[b]
	d_cCOM_GTP_S2	$1.78 \leq x \leq 1.85$ nm	
S2-open	d_gly12_gly60	$0.6 \leq x \leq 5.0$ nm	
	d_gly12_{61}*	$0.8 \leq x \leq 5.0$ nm	
	d_GTP_glu62	$1.0 \leq x \leq 5.0$ nm	*and*[b]
	d_GTP_glu63	$1.0 \leq x \leq 5.0$ nm	
	d_cCOM_GTP_S2	$1.975 \leq x \leq 5.0$ nm	

[*] {61} is glutamine 61 for the wild type and leucine 61 for the Q61L mutant. [a] One or more of the conditions must be true. [b] All conditions must be true.

The State column are the names of the stable states. Every stable state is build by combining the Constraints and Logic columns. For example in set notation the S2-GTP state corresponds to $((\{\, x \mid \text{d\_gly12\_gly60}(x) \in [0.0, 0.3] \,\} \cup \{\, x \mid \text{d\_gly12\_\{61\}}(x) \in [0.0, 0.3] \,\} \cup \{\, x \mid \text{d\_GTP\_glu62}(x) \in [0.0, 0.65] \,\} \cup \{\, x \mid \text{d\_GTP\_glu63}(x) \in [0.0, 0.65] \,\}) \cap \{\, x \mid \text{d\_cCOM\_GTP\_S2}(x) \in [0.0, 1.6] \,\})$ in words this would be: $((0.0 \leq \text{d\_gly12\_gly60}(x) \leq 0.35$ *or* $0.0 \leq \text{d\_gly12\_\{61\}}(x) \leq 0.35$ *or* $0.0 \leq \text{d\_GTP\_glu62}(x) \leq 0.65$ *or* $0.0 \leq \text{d\_GTP\_glu63}(x) \leq 0.65)$ *and* $0.0 \leq \text{d\_cCOM\_GTP\_S2}(x) \leq 1.6)$

Appendix B: Transitions as function of the Monte-Carlo (MC) steps The figures listed in this appendix show the type of transition as sampled for each step in the TPS simulations. The x-axes represent the number of the MC trials, while the y-axis shows the sampled transition. The y-axis lists the transitions, ordered such that the simulation can only switch to the transitions directly above or below the current transition, or between the top and bottom transition.

Throughout most of the WT simulations, switching occurs on average every 16 MC steps, indicating that the simulation loses memory of the starting transition path. The second part of the simulation starting from an S2-α3 to S2-open transition is an exception, as this simulation remains in the S2-open $\rightarrow$ S2-GTP transition for over 1000 MC steps. For the S2 Q61L simulations the accepted and decorrelating MC steps are also distributed uniformly throughout all three simulations. All simulations spend a significant amount of simulation steps in the S2-open $\rightarrow$ S2-α3 transition, possibly indicating that the barrier separating the open state from the S2-α3 state is lower in Q61L.

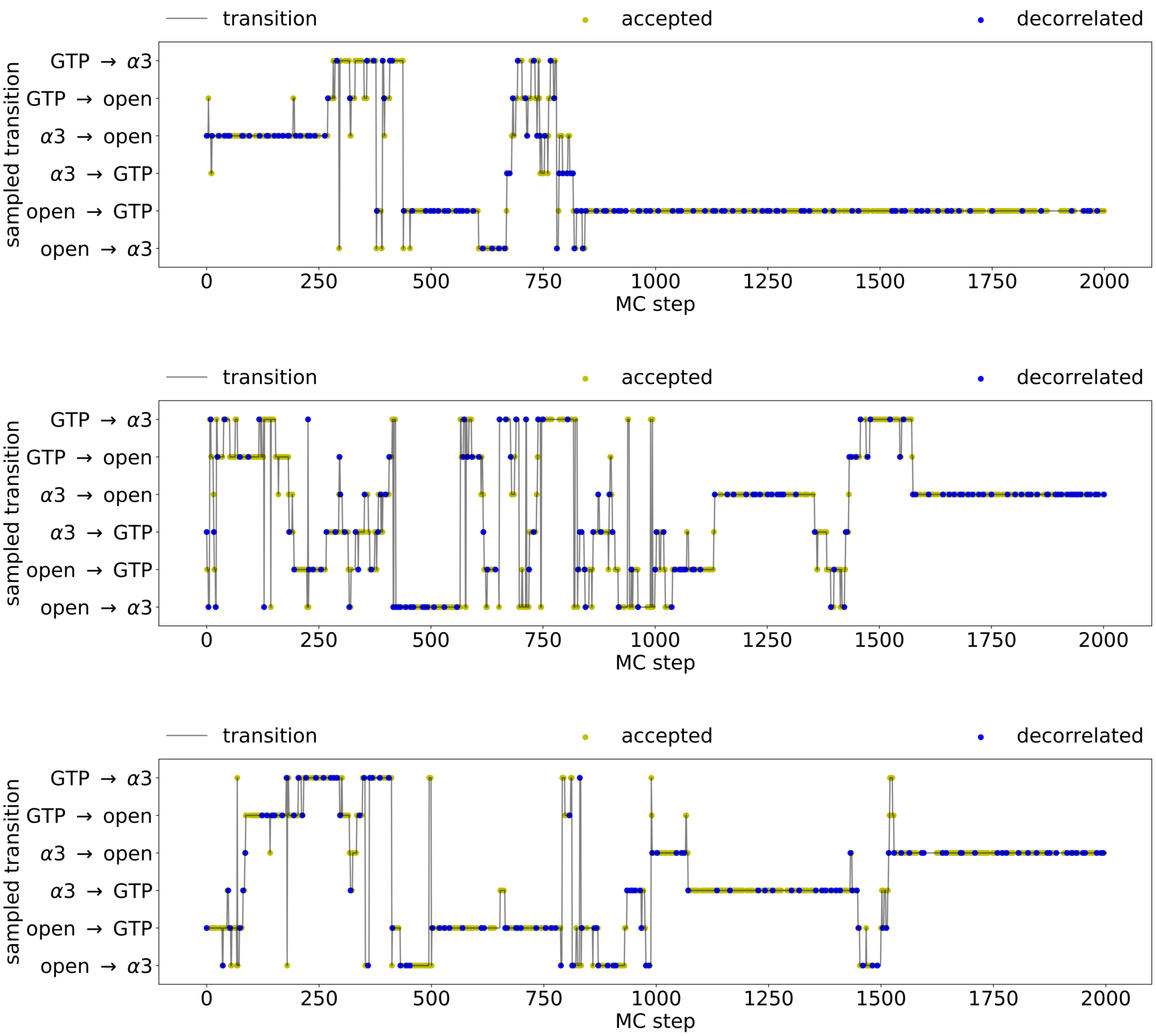

Figure 1: **Transitions as function of the Monte-Carlo step for the WT simulations.** The simulations started from (top) the S2-α3 to S2-open transition, (middle) the S2-α3 to S2-GTP transition and (bottom) the S2-open to S2-GTP transition. The x-axis shows the number of the MC steps. The y-axis shows the sampled transition. The y-axis lists all transitions that can occur for S2. The gray lines represents the trial moves, with the accepted MC steps highlighted as yellow dots and the accepted MC steps that lead to a new decorrelated trajectory with a blue dot.

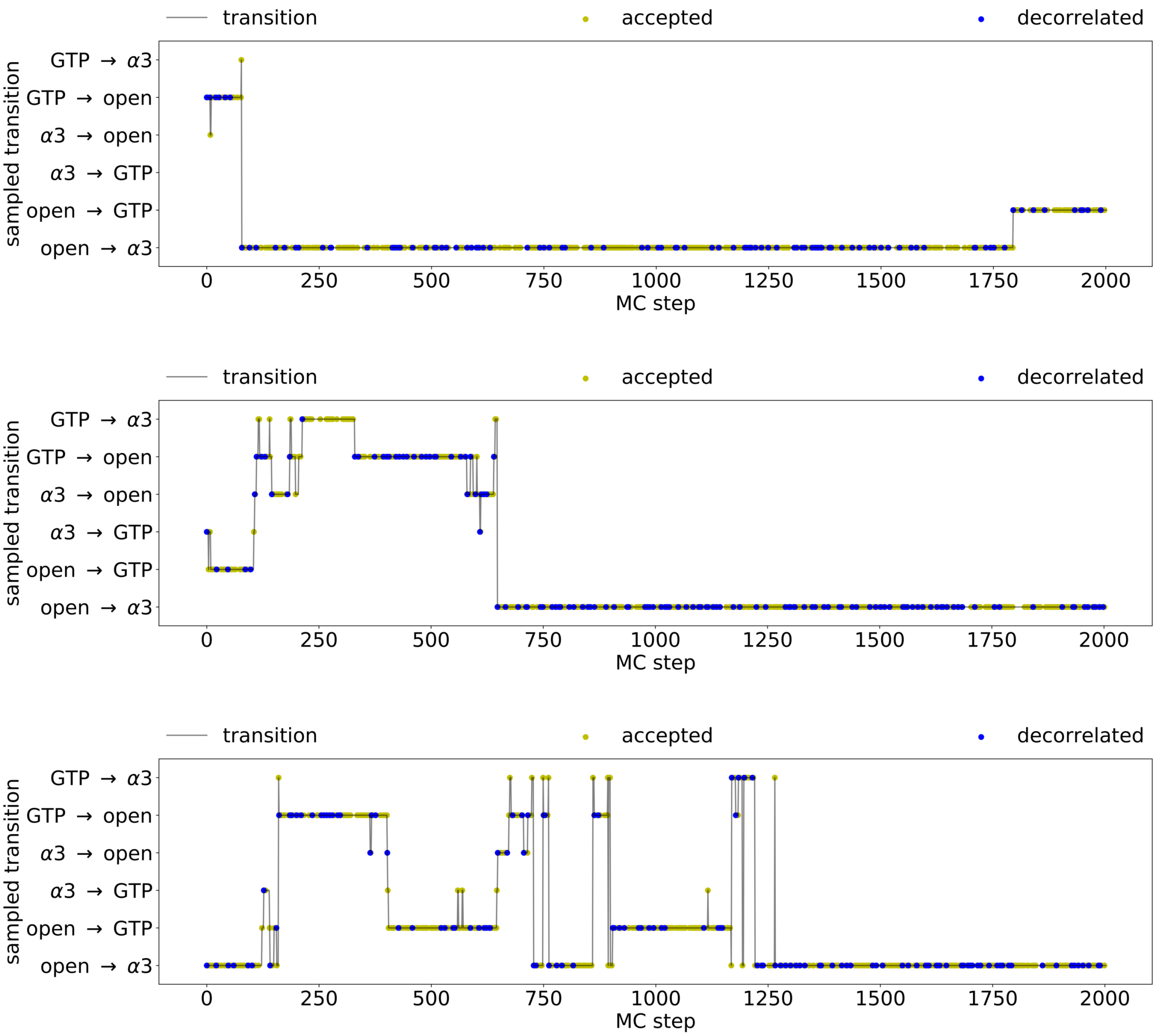

Figure 2: **Transitions as function of the Monte-Carlo step for the Q61L simulations.** The simulations started from (top) the S2-GTP to S2-open transition, (middle) the S2-$\alpha 3$ to S2-GTP transition and (bottom) the S2-open to S2-$\alpha 3$ transition. The x-axis shows the number of the MC steps. The y-axis shows the sampled transition. The y-axis lists all transitions that can occur for S2. The gray lines represents the trial moves, with the accepted MC steps highlighted as yellow dots and the accepted MC steps that lead to a new decorrelated trajectory with a blue dot.

Appendix C: MSTPS results for S2 The sampling statistics of the S2 MSTPS simulations are shown in table 1 in the main text. The number of Monte Carlo (MC) trials was equal for all simulations. The acceptance is between 34 % and 42 %, which is reasonable considering the theoretical maximum of 67 %. This theoretical maximum is due to the fact that in our shooting algorithm only self transitions are forbidden. This leads to a maximum acceptance of $\frac{(N-1)}{N}$, for N number of states. With $N = 3$ this leads to the theoretical maximum acceptance of 67% for this MSTPS study. The number of decorrelated trajectories is satisfactory for all simulations, and are spread well throughout the simulation as shown by the blue dots in the figures in Appendix B. The average path length and total simulation time are only different for WT simulation 1. This simulation enters a different transition channel than the other simulations, which would explain these altered numbers.

The Least Changed Path (LCP) connects parts of accepted paths between backward and forward shooting points, and provides insights into the conformations of the system on top of the barrier. This concept is best explained with an example: From a starting path, a new path is generated with a forward shot. The new path contains part of the original path, and part that is newly generated. Then, another path is generated from a backward shot, taken from the newly generated part. And then, yet another new path is generated, from a forward shot, taken from the newly generated part. Connecting the parts of the paths between the forward shot, backward shot and again forward shot is called the least changed path .[3] Figure 3 (WT) and figure 4 (Q61L) show the LCPs for all simulations, projected on top of the combined pdhs from figure 7in the main text. The colouring is based on the first sampled transition of each frame of the LCP and is red for S2-GTP $\leftrightarrow$ S2-open, blue for S2-GTP $\leftrightarrow$ S2-α3, and yellow for S2-α3 $\leftrightarrow$ S2-open. For the WT, all transitions sample the same diffuse barrier region, as indicated by the overlap of the clouds, which supports the hypothesis that the switching is also a diffusive process. For the extra channel for the S2-GTP$\leftrightarrow$S2-open transition, this mostly occurs in simulation 1 of WT, but it is also observed in simulation 2 and 3. For the Q61L simulations, the LCP is more constrained to a

value of under 1.3 nm for the S2-α3-distance. Also, the clouds overlap less with each other, making switching more unlikely. Simulation 1 and 3 of Q61L also show sampling of an extra S2-α3 $\leftrightarrow$ S2-open channel, at values of 0.75 nm or lower for the S2-α3-distance.

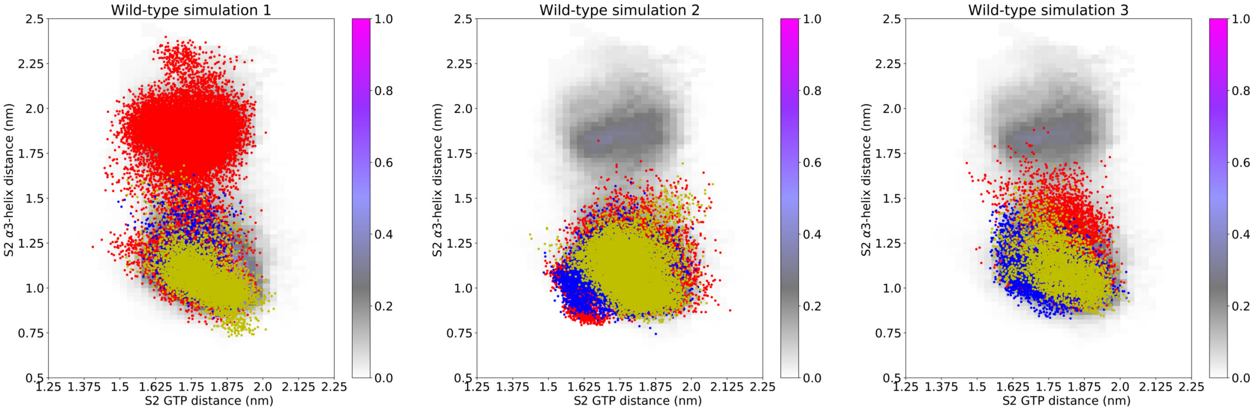

Figure 3: **The Least-Changed-Paths of the WT simulations.** The frames of the LCP of each WT simulation, shown on top of the combined path density histogram, as shown in (figure 7(top) in the main text). The color of each frame represents the first transition sampled by that frame, red for S2-GTP $\leftrightarrow$ S2-open, blue for S2-GTP $\leftrightarrow$ S2-α3, and yellow for S2-α3 $\leftrightarrow$ S2-open. The numbering of the simulations is in the order of figure 1.

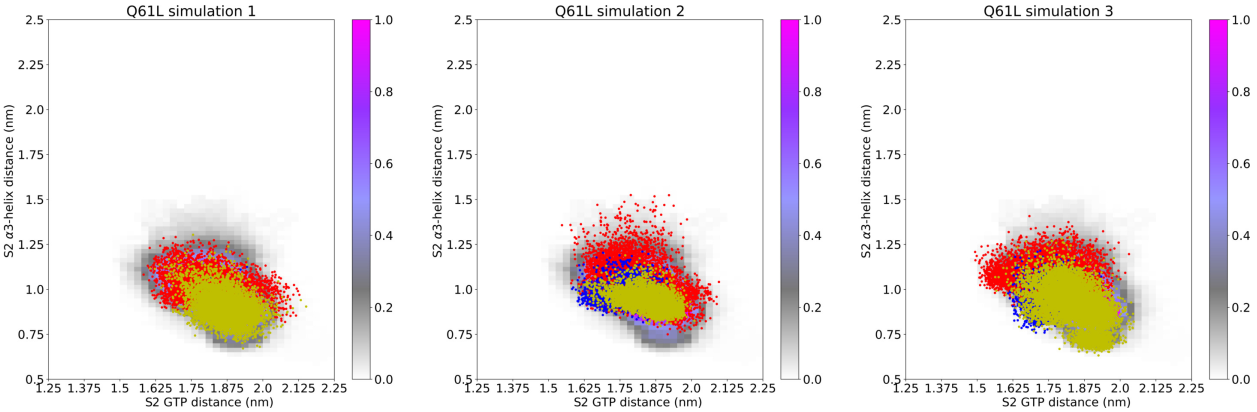

Figure 4: **The Least-Changed-Paths of the Q61L simulations**. The LCP are shown on top of the combined path density histogram (figure 7(top) in the main text). The color of each frame represents the first transition sampled by that frame, and is the same as in figure 3. The numbering of the simulations is in the order of figure 2.

Visual inspection of the transition paths as sampled for WT shows that in some paths helix α2 (residues 65-73) contained within S2, unfolds when entering the open state, but retains its shape in the S2-open state for the Q61L mutant. Two-dimensional probability

histograms of the S2-$\alpha3$ distance and the number of helical hydrogen bonds of the $\alpha2$-helix (residues 65-73), for frames in the S2-open state, are shown in figure 5 for both WT and Q61L. Looking at the WT plot, there are two maxima for states in the reaction channel close to the $\alpha3$-helix (under 1.5 nm on the y-axis), one where the $\alpha2$-helix has all 5 helical H-bonds and one where it has only 1 helical H-bond. For the WT S2-open states away from the $\alpha3$-helix (above 1.5 nm on the y-axis), the $\alpha2$-helix has lost part of it helical structure, as indicated by a distribution around 2 helical H-bonds. When looking at the transition region between these two reaction channels at around 1.5 on the y-axis, helix $\alpha2$ has lost most of its helical hydrogen bonds, which may indicate a correlation between the unfolding of helix $\alpha2$ and the switching between the two reaction channels. The probability histogram for the S2-open frames of Q61L show a maximum at 4 helical H-bonds and S2 close to helix $\alpha3$. These observations suggest that Q61L has a more structured open state.

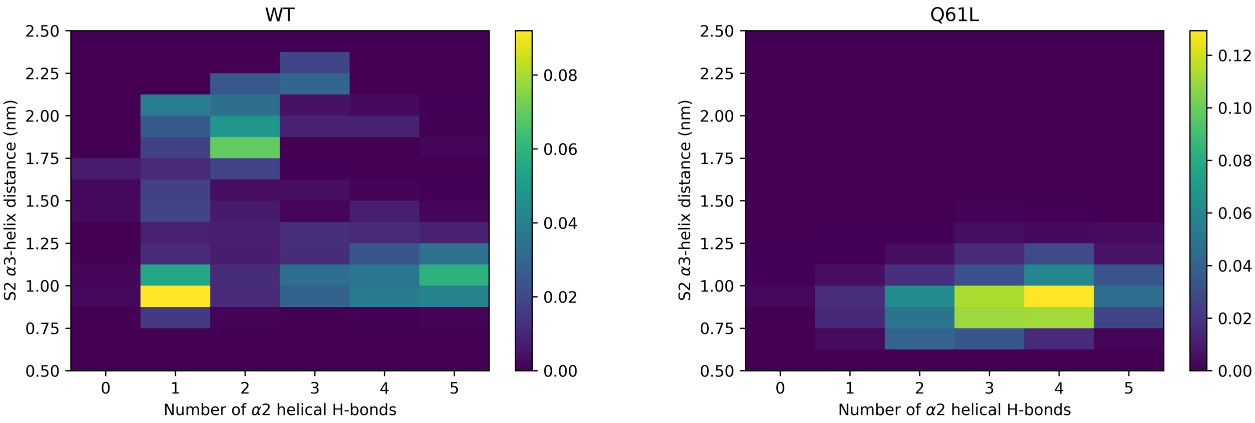

Figure 5: **Two-dimensional probability histogram of the S2-$\alpha3$-helix distance and the number of helical hydrogen bonds in $\alpha2$-helix for the S2-open state.** These are shown for (left) WT and (right) Q61L. The y-axis is the cCOM of S2 to the $\alpha3$-helix (as used in figure 7in the main text). The x-axis is the number of hydrogen bonds (as described in table 1) between the backbone O of residue i and the backbone NH of residue $i + 4$ for $i \in [65, 69]$. The colors indicate the probability.

Appendix D: MSTPS results for S1 The transitions as function of the MC trials of the S1 simulations are shown in figure 6. The x-axes represent the number of the MC trials, while the y-axis shows the sampled transition. The y-axis lists the transitions, ordered

such that the simulation can only switch to the transitions directly above or below the current transition, or between the top and bottom transition, similar to the figures shown in Appendix B. The accepted and decorrelating MC steps are distributed well throughout both simulations. The number of switches that occur between the transitions is similar for both WT and Q61L. Both simulations spend a significant amount of simulation steps in the 30-32 → open transition.

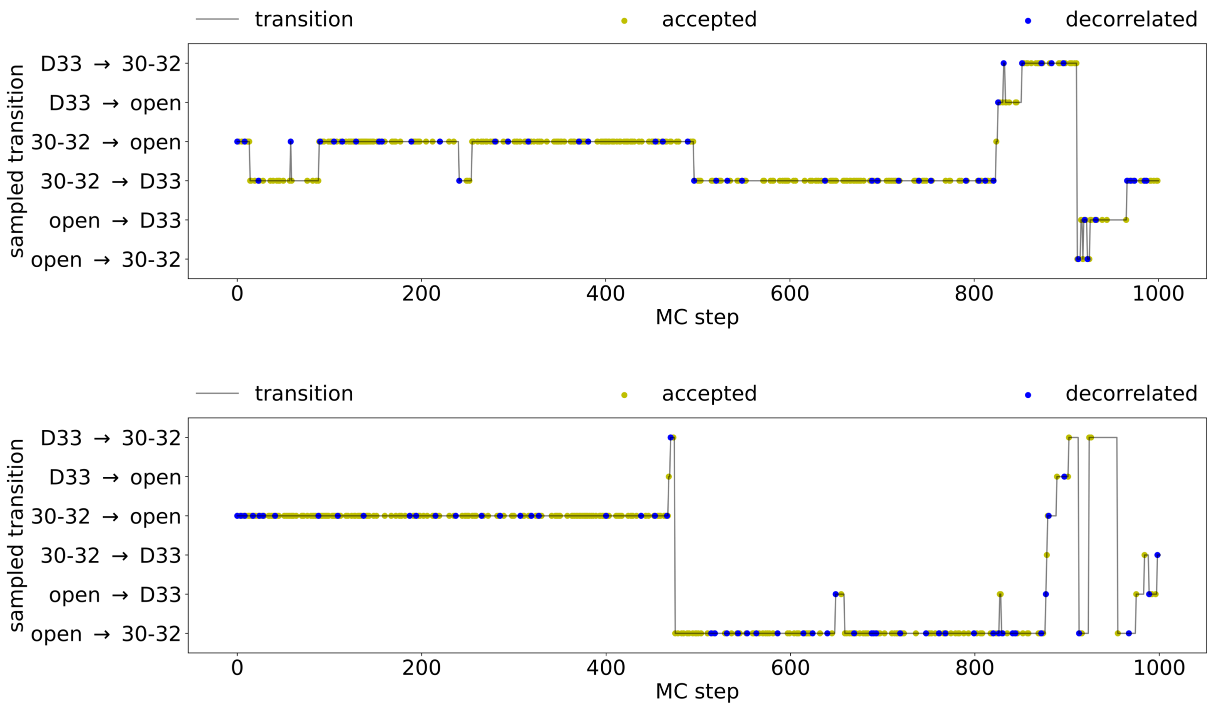

Figure 6: **Transitions as function of the Monte-Carlo step for the S1 simulations.** (top) WT (bottom) Q61L. The same axis setup and labeling is used as in the supplements for figures 1 and 2. Here D33 corresponds to the S1-D33 state, 30-32 to the S1-30-32 state, and open to the S1-open state.

References

(1) McGibbon, R. T.; Beauchamp, K. A.; Harrigan, M. P.; Klein, C.; Swails, J. M.; Hernández, C. X.; Schwantes, C. R.; Wang, L.-P.; Lane, T. J.; Pande, V. S. MDTraj: A Modern Open Library for the Analysis of Molecular Dynamics Trajectories. *Biophysical Journal* **2015**, *109*, 1528 – 1532.

(2) Bai, L.; Breen, D. Calculating Center of Mass in an Unbounded 2D Environment. *Journal of Graphics Tools* **2008**, *13*, 53–60.

(3) Juraszek, J.; Vreede, J.; Bolhuis, P. G. Transition path sampling of protein conformational changes. *Chemical Physics* **2012**, *396*, 30–44.
